# Identification of the key immune-related genes and immune cell infiltration changes in renal interstitial fibrosis

**DOI:** 10.3389/fendo.2023.1207444

**Published:** 2023-11-08

**Authors:** Zhitao Dong, Fangzhi Chen, Shuang Peng, Xiongfei Liu, Xingyang Liu, Lizhe Guo, E. Wang, Xiang Chen

**Affiliations:** ^1^ Department of Urology, The Second Xiangya Hospital, Central South University, Changsha, Hunan, China; ^2^ Department of Anesthesiology, Xiangya Hospital, Central South University, Changsha, Hunan, China; ^3^ National Clinical Research Center for Geriatric Disorders (Xiangya Hospital), Central South University, Changsha, Hunan, China

**Keywords:** chronic kidney disease, renal interstitial fibrosis, immune cells, biomarker, NF-κB signaling pathway

## Abstract

**Background:**

Chronic kidney disease (CKD) is the third-leading cause of premature mortality worldwide. It is characterized by rapid deterioration due to renal interstitial fibrosis (RIF) via excessive inflammatory infiltration. The aim of this study was to discover key immune-related genes (IRGs) to provide valuable insights and therapeutic targets for RIF in CKD.

**Materials and methods:**

We screened differentially expressed genes (DEGs) between RIF samples from CKD patients and healthy controls from a public database. Least absolute shrinkage and selection operator regression analysis and receiver operating characteristic curve analysis were applied to identify significant key biomarkers. The single-sample Gene Set Enrichment Analysis (ssGSEA) algorithm was used to analyze the infiltration of immune cells between the RIF and control samples. The correlation between biomarkers and immune cell composition was assessed.

**Results:**

A total of 928 DEGs between CKD and control samples from six microarray datasets were found, 17 overlapping immune-correlated DEGs were identified by integration with the ImmPort database, and six IRGs were finally identified in the model: apolipoprotein H (APOH), epidermal growth factor (EGF), lactotransferrin (LTF), lysozyme (LYZ), phospholipid transfer protein (PLTP), and secretory leukocyte peptidase inhibitor (SLPI). Two additional datasets and *in vivo* experiments indicated that the expression levels of APOH and EGF in the fibrosis group were significantly lower than those in the control group, while the expression levels of LTF, LYZ, PLTP, and SLPI were higher (all P < 0.05). These IRGs also showed a significant correlation with renal function impairment. Moreover, four upregulated IRGs were positively associated with various T cell populations, which were enriched in RIF tissues, whereas two downregulated IRGs had opposite results. Several signaling pathways, such as the “T cell receptor signaling pathway” and “positive regulation of NF-κB signaling pathway”, were discovered to be associated not only with immune cell infiltration, but also with the expression levels of six IRGs.

**Conclusion:**

In summary, six IRGs were identified as key biomarkers for RIF, and exhibited a strong correlation with various T cells and with the NF-κB signaling pathway. All these IRGs and their signaling pathways may evolve as valuable therapeutic targets for RIF in CKD.

## Introduction

Chronic kidney disease (CKD) is increasingly recognized as a serious public health epidemic worldwide, with a high incidence rate, high medical costs, and poor outcomes (1, 2). According to the GBD 2017 estimates, approximately 697.5 million cases were diagnosed in 2017, which was estimated to be 9.1% of the global population (3). Importantly, CKD directly causes 1.2 million deaths and an additional 1.4 million deaths from cardiovascular disease resulting from impaired kidney function, making CKD the 12^th^ leading cause of death worldwide (3). CKD is usually caused by many conditions that put a strain on the kidneys, including glomerulonephritis, diabetes, high blood pressure, hereditary nephropathy, and renal tubulointerstitial disease (4, 5). However, renal fibrosis, particularly renal interstitial fibrosis (RIF), is the final common pathological outcome of almost all advanced kidney diseases, whatever the original etiology (6). Despite many promising clinical studies and experimental data, currently available treatment strategies can only ameliorate or delay the progression of CKD rather than reverse the renal fibrosis (5, 6). Therefore, there is an urgent need to conduct mechanistic research on renal fibrosis in CKD to understand the underlying pathogenesis of the process and to optimize treatment strategies, letting us improve the prognosis of patients with fibrotic kidney disorders.

RIF mainly manifests as sclerosis, tubular atrophy, and inflammatory infiltration, resulting in a dynamic, multifactorial process, including fibroblast activation, tubular epithelial-to-mesenchymal transition (EMT), and T-cell and monocyte/macrophage infiltration (7). The initial step of renal fibrosis is uncontrolled or excessive inflammatory infiltration, which is a key factor that cross-talks with the progression of subsequent series of fibrosis (8). Recently, antifibrotic agents that target inflammation have been recommended as potential alternative therapies for renal fibrosis. As a general immunosuppressive cytokine, IL-10 delivered by hydrogels can reduce macrophage infiltration and apoptosis to treat renal fibrosis and chronic kidney disease (9). Similarly, IκB (an inhibitor of NF-κB) treatment and β2 adrenergic receptor agonists both inhibit NF-κB signaling and result in the inactivation of macrophages, thereby inhibiting further kidney damage (10, 11). However, these ongoing and completed clinical trials still lack sufficient evidence for successful targeted fibrosis in CKD (12). Now one opinion considers that the intracellular signaling pathways associated with fibrosis interact with other signaling transductions that affect critical cellular activation and functions. Given that inflammation is self-sustaining and multifactorial, identifying the key genes associated with inflammation and describing the interaction with the enrichment of immune cells will be significant in elaborating the potential mechanisms of RIF in CKD.

Several evidences indicating that chronic or unresolved inflammation plays a pivotal role in the onset and progression of renal fibrosis (7, 8). Inflammatory cells, such as macrophages and T lymphocytes, infiltrate the renal interstitium and continually secrete pro-inflammatory cytokines. These substances activate fibroblasts and contribute to maladaptive repair processes and progressive renal fibrosis. It has been observed that T cells polarized towards a Th2 phenotype can induce fibroblast activation and promote alternative activation of macrophages, potentially fostering renal fibrosis (13). Moreover, infiltrating immune cells locally produce TGF-β, which further amplifies renal fibrosis and inflammation by activating various signaling molecules (AKT/mTOR, Smad2/3, NF-κB, KLF6, and Sp1) (12). Recently discovered inflammation-related biomarkers and distinct patterns of immune infiltration in RIF can offer additional insights into the risk associated with fibrosis in CKD (14). However, since renal fibrosis represents a common pathological manifestation of chronic kidney diseases with diverse causes, studies based on isolated causal samples or limited analytical dimensions may introduce biases.

Therefore, we downloaded six microarray datasets from renal interstitial tissues of CKD patients with different causes to screen coexpressed differentially expressed genes (co-DEGs) and identified key immune-related genes (IRGs) for RIF using the least absolute shrinkage and selection operator (LASSO) regression analysis between RIF and healthy control samples, which verified with *in vivo* experiments. Next, correlation analysis was conducted between the expression level of IRGs and the infiltration of immune cells which obtained by the single-sample gene set enrichment analysis (ssGSEA) method. Kyoto Encyclopedia of Genes and Genomes (KEGG) analysis and GSEA were also performed to discover the biological function and significant signaling pathways correlated with RIF. Through a multidimensional analysis of the interrelationship between IRGs and immune cells, as well as potential pathway correlation analyses, our study aims to uncover key immune-related biomarkers and provide novel insights into the potential immune mechanisms associated with the progression of RIF.

## Materials and methods

### Patient cohort and data preparation

The discovery cohorts of the study, including GSE30529, GSE35487, GSE37455, GSE133288, GSE121211, and GSE32591 datasets, were downloaded from the Gene Expression Omnibus (GEO) website (https://www.ncbi.nlm.nih.gov/geo) for DEG analysis. All available datasets included renal interstitial samples from healthy controls and patients with CKD. Those CKD patients were mostly diagnosed with diabetic nephropathy (DN), hypertensive nephropathy, IgA nephropathy, membranous nephropathy (MN), minimal change disease (MCD), focal and segmental glomerulosclerosis (FSGS), or systemic lupus erythematosus (SLE).

The GSE12682 dataset was downloaded and analyzed to identify the key markers associated with RIF. A total of 36 renal tubulointerstitial samples, including 23 CKD samples with evidence of tubulointerstitial fibrosis and 13 healthy control samples, were enrolled in the GSE12682 dataset. In addition, the GSE76882 dataset was analyzed for external validation to examine its key gene signature. It included 99 healthy controls, 42 interstitial fibrosis and tubular atrophy (IFTA) samples, 11 IFTA with inflammation (IFTA-i) samples, and 29 IFTA with acute rejection (IFTA-AR) samples. Given that we aimed to investigate the mechanisms underlying fibrosis rather than the posttransplant immune response, we selected healthy controls and IFTA samples for further analysis. In addition, the GSE38117 dataset, which studied renal fibrosis using experimental model of ureteral unilateral obstruction (UUO) in three mice, was also used to validate the key markers associated with RIF. Surgery was performed by complete ligation of the left ureter, which the control lateral right kidney served as internal control. The basic information for the included datasets is shown in **Supplementary Table 1**.

All the gene expression profiling data were first subjected to background correction and quartile normalization of the raw data using the “Limma” package of R, followed by batch effect elimination using the “sva” package, to obtain normally distributed expression values. The DEGs between CKD samples and healthy control samples were those that had absolute value of log2 fold change (|logFC|) > 1 and adjusted *P* value < 0.05. Then, the robust rank aggregation (RRA) method was used to integrate and identify overlapping DEGs (*P* value < 0.05) from the discovery cohorts.

### Identification of immune-related DEGs

To identify the immune-related DEGs (IRGs), we downloaded a total of 1793 immune-related genes from the Immunology Database and Analysis Portal (ImmPort) website (https://www.immport.org). These immune-related genes originated from 17 immune-related categories, including antigen processing and presentation, antimicrobials, and the BCR signaling pathway. Then, we integrated the co-DEGs from the discovery cohorts and immune-related gene sets and identified the overlapping IRGs for further analysis.

### Exploration of key IRGs

To explore the key genes in IRGs associated with RIF, LASSO algorithm was applied for all renal tubulointerstitial samples from the GSE12682 dataset using the “glmnet” package in R. LASSO regression is a type of linear regression that uses shrinkage to regularize regression algorithms. Regularization can solve the overfitting problem by adding more parameters, leaving fewer parameters in the model, and limiting its complexity. L1 regularization was executed by adding a penalty equal to the absolute value of the magnitude of each coefficient in the LASSO regression model. This type of regularization contributes to constructing sparse models with relatively few coefficients. Those variables whose coefficients are zero are removed from the model. Therefore, we calculated the sum of the candidate gene values multiplied by the corresponding coefficient obtained from LASSO regression analysis, and named it as risk score.

### Validation of the risk score model

To evaluate the value of the risk score model, the GSE12682 dataset (training set) and the GSE76882 dataset (validation set) were used to validate the accuracy and diagnostic ability of the risk score model in CKD patients with RIF via the “pROC” package in R. We visualized the area under the curve (AUC) of the ROC curve by calculating the sensitivity and specificity values with the “pROC” package. Then calibration curve was also used to visualize the performance of the risk score model with the Hosmer-Lemeshow test using the “ResourceSelection” package in R. The Hosmer–Lemeshow test is used frequently to calculate the goodness of fit of risk prediction models, in which a *P* value > 0.05 indicates that the data fit by the risk prediction model are at an acceptable level and that the scoring model works well.

### Gene set enrichment analysis

Through the DAVID tools (http://david.ncifcrf.gov/), Gene Ontology (GO) and KEGG analyses were used to discover the biological function and significant signaling pathways correlated with RIF. The results were visualized via the “clusterProfiler” R package. We applied strict cutoff values of false discovery rate (FDR) < 0.05 and adjusted *P* value < 0.05 to detect statistically significant GO terms and KEGG pathways. GSEA was used to discover the functional terms associated with RIF with the thresholds of an NOM *P* value < 0.05 and |NES| > 1. Each analysis was performed with 1000 times of arrangements of the gene set.

### Discovery of immune cell subtypes and correlation with key biomarkers

To quantify the relative infiltration of immune cells for each sample, the ssGSEA algorithm was performed to calculate the normalized enrichment scores of 28 types of immune cells in the RIF samples and control samples using the Gene Set Variation Analysis (GSVA) R package (15). In detail, expression data of renal interstitial samples between the RIF and healthy control groups were used to calculate immune cell abundances according to the GSVA algorithm and specific cell markers (**Supplementary Table 2**) (16). The 28 immune cells were gamma/delta T cells, activated CD4 T cells, activated CD8 T cells, activated dendritic cells (DCs), central memory CD8 T cells, central memory CD4 T cells, effector memory CD4 T cells, effector memory CD8 T cells, monocytes, macrophages, immature DCs, immature B cells, activated B cells, memory B cells, mast cells, eosinophils, myeloid-derived suppressor cells (MDSCs), CD56dim natural killer cells, CD56bright natural killer cells, natural killer cells, natural killer T cells, neutrophils, plasmacytoid DCs, regulatory T (Treg) cells, T follicular helper cells, type 1 T helper cells, type 17 T helper cells, and type 2 T helper cells. Violin plot was used to visualize the differences in the composition of 28 immune cell subtypes between the RIF samples and healthy control samples with the two-sided Wilcoxon test. We also conducted Pearson correlation analysis between key IRGs and immune cell markers in the human kidney, which were obtained from the CellMarker database (http://biocc.hrbmu.edu.cn/CellMarker/), as well as gene sets of specific signaling pathways from the KEGG database (https://www.genome.jp/kegg/).

### Clinical correlation analysis

In addition, we also used the Nephroseq V5 tool (http://v5.nephroseq.org/) to identify the difference in risk score calculated by the expression level of key IRGs between CKD and control samples and explored the correlation between the risk score and clinical indices of renal function, including glomerular filtration rate (GFR), proteinuria, blood urea nitrogen (BUN), and serum creatinine level (SCR), in CKD patients using Pearson correlation analysis. In the Nephroseq tool, we downloaded the clinical data of CKD patients in the GSE104954 dataset and GSE30529 dataset.

### Renal interstitial fibrosis model establishment *in vivo*

To verify the key value of 6 IRGs *in vivo*, renal stone model was established by adding 1% ethylene glycol (EG) (324558, Sigma-Aldrich, USA) in drinking water for 4 weeks, as described in our previous study (17). The animal experiment was approved by the Ethics Committee for Animal Research of the Xiangya hospital of Central South University (202301003).

A total of 10 male Sprague-Dawley (SD) rats (age: 6-8 weeks, weight: 250-300 g) were purchased from the Laboratory Animal Center of Central South University (Changsha, China), and were housed in the controlled condition (12 h light/dark cycle, humidity (40-60%) and steady temperature of 22 ± 0.5°C) with free access to water and food. The rats were randomly divided into the control group and stone model group (n = 5 per group). In the stone model group, the rats received drinking water containing ethylene glycol (1%) for 4 weeks, while the rats in the control group had access to normal drinking water without ethylene glycol for 4 weeks. All rats were sacrificed by cervical dislocation under anesthesia [pentobarbital sodium (40 mg/kg)] after 4 weeks intervention. Kidney tissues were collected at -80°C or fixed in paraformaldehyde or formalin solution for histological study. Hematoxylin and eosin (HE) and Sirius Red staining were used to visualize the RIF in the rat with renal stone model. Finally, the stained area was observed and photographed with a bright-field microscope. Two experienced pathologists examined the extent of kidney injury. Kidney injury scores were evaluated using a scale ranging from 0 to 4 (0 indicating normal; 1 representing less than 25%; 2 indicating 25-50%; 3 representing 50-75%; and 4 indicating greater than or equal to 75%). Sirius Red positive area was quantified using Image J software (NIH Image, Bethesda, MD).

### Quantitative real-time PCR

Total RNA was extracted from renal tissue using a Total RNA Kit II (R6934-01; Omega Bio-tek, Norcross, GA, USA) following the manufacturer’s instructions. Then, cDNA was synthesized using an RT Reagent Kit with gDNA Eraser (No. RR047A; Takara, Tokyo, Japan). The mRNA levels of 6 IRGs were detected using All-in-OneTM qPCR Mix (No: QP001; GeneCopoeia, Germantown, MD, USA). The primer sequences are listed in **Supplementary Table 3**. Each sample was repeated three times.

### Statistical analysis

The present study performed all statistical analyses using R software (Version 4.1.1; R Foundation for Statistical Computing, Vienna, Austria). The Wilcoxon test was run on expression data, with visualization by two groups of boxplots. Correlations among the expression levels of different genes were evaluated by Pearson correlation coefficients and were visualized using the “corrplot” package. A two-sided *P* value < 0.05 was accepted as statistically significant.

## Results

### Screening of differentially expressed genes

**Figure 1:**
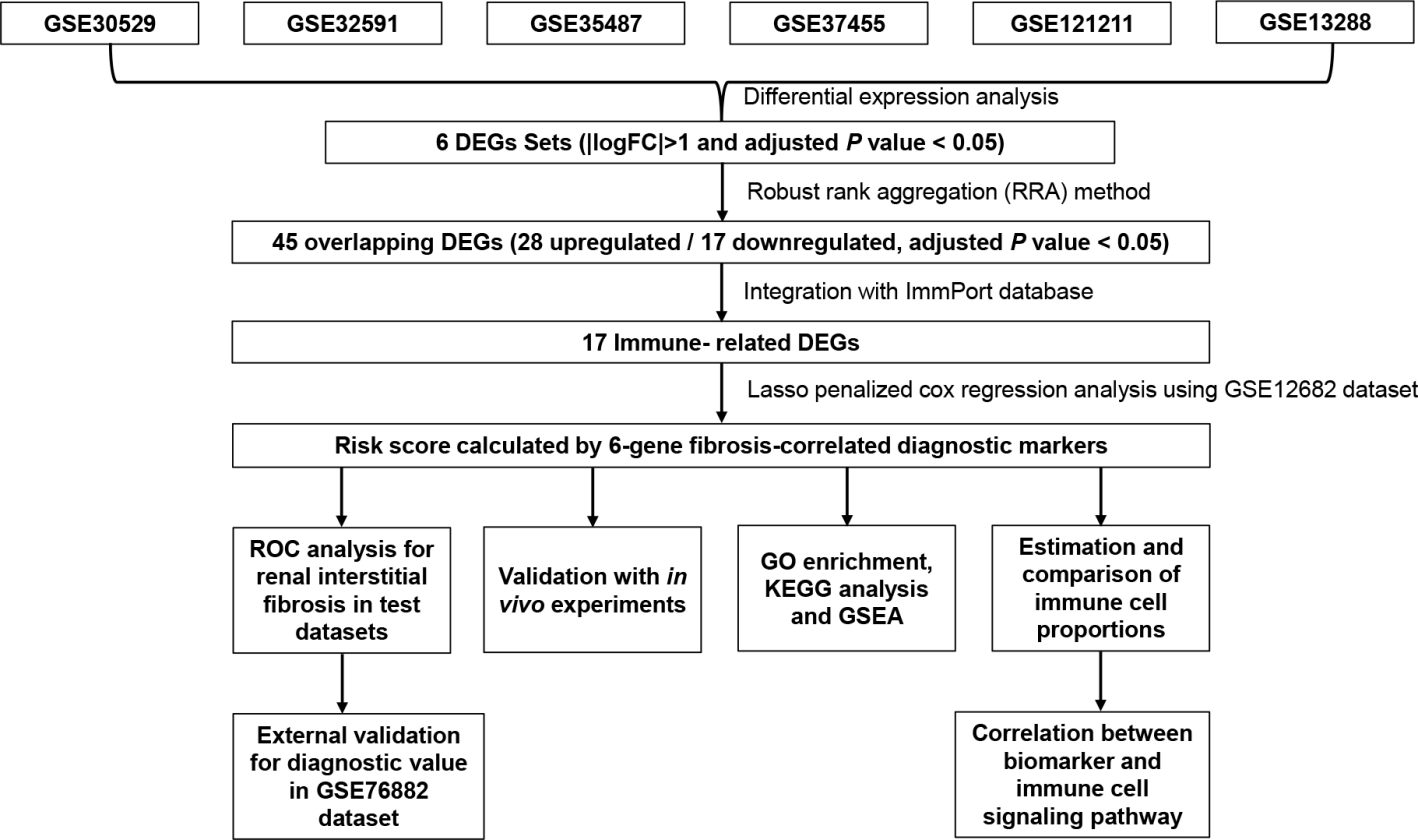
Flowchart describing the process used to identify and validate the key biomarkers of renal interstitial fibrosis. DEGs, differentially expressed genes; ROC, receiver operating characteristic.

The overall flow chart of the study design is presented in **Figure 1**. Given that RIF mostly results from the progression of CKD, we downloaded and analyzed six microarray expression datasets from individuals with CKD and healthy controls, including the GSE30529, GSE32591, GSE35487, GSE37455, GSE121211, and GSE133288 datasets. Of the DEGs between the CKD and control samples, 416 DEGs were identified from the GSE30529 dataset, including 298 upregulated and 118 downregulated genes (**Supplementary Figure 1A**). A total of 125 DEGs (including 97 upregulated and 28 downregulated genes) were screened from the GSE32591 dataset (**Supplementary Figure 1B**), 36 DEGs (including 0 upregulated and 36 downregulated genes) from the GSE35487 dataset (**Supplementary Figure 1C**), 27 DEGs (including 10 upregulated and 17 downregulated genes) from the GSE37455 dataset (**Supplementary Figure 1D**), 78 DEGs (including 49 upregulated and 29 downregulated genes) from the GSE121211 dataset (**Supplementary Figure 1E**), and 246 DEGs (including 48 upregulated and 198 downregulated genes) from the GSE133288 dataset (**Supplementary Figure 1F**).

### Identification of immune-correlated key markers

**Figure 2:**
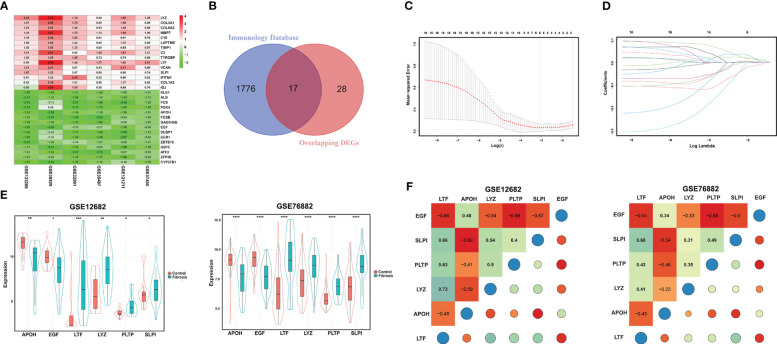
Identification of DEGs for RIF. **(A)** Heatmap of each expression microarray. The heat map of the top 15 upregulated (red) and downregulated (green) DEGs identified by the robust rank aggregation method applied to the six microarray datasets. The value in each column is the LogFC. **(B)** Venn diagram of overlapping DEGs and a set of immune-related genes from the Immunology Database. **(C, D)** LASSO deviance profiles and LASSO coefficient profiles. **(E)** The expression levels of the six key IRGs identified by LASSO regression analysis between the RIF and control samples in the GSE12682 and GSE76882 datasets. **(F)** Heatmap of correlations for the six key IRGs in the GSE12682 dataset (left) and GSE76882 dataset (right). The size of the colored squares and circles represents the strength of the correlation. Darker color implies a stronger association. *, *P* < 0.05; **, *P* < 0.01; ***, *P* < 0.001; ****, *P* < 0.0001. RIF, renal interstitial fibrosis; DEGs, differentially expressed genes; IRGs, immune-related DEGs.

After screening the DEGs from the six datasets, we integrated those DEG sets using the RRA method, and 45 overlapping DEGs (including 28 upregulated and 17 downregulated genes) were identified. The top 15 overlapping upregulated and downregulated DEGs in the six datasets are shown in **Figure 2A**. Then, we matched these 45 DEGs with immune-related gene sets from the ImmPort database and found 17 overlapping IRGs (**Figure 2B**). To identify the key markers associated with RIF, we performed LASSO regression analysis with those 17 IRGs for the GSE12682 dataset, which contained 23 CKD samples with evidence of tubulointerstitial fibrosis and 13 healthy control samples, and finally identified six IRGs in the model: apolipoprotein H (APOH), epidermal growth factor (EGF), lactotransferrin (LTF), lysozyme (LYZ), phospholipid transfer protein (PLTP), and secretory leukocyte peptidase inhibitor (SLPI) (**Figures 2C, D**).

**Figure 3:**
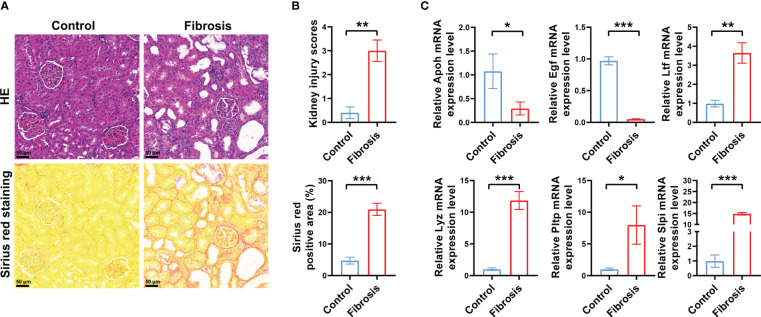
Verification of DEGs *in vivo*. **(A)** Representative images of HE and Sirius red staining of RIF in the control group and renal fibrosis group with renal stone model (200×). **(B)** Quantitative analysis of HE and Sirius red staining in kidneys of RIF in the control group and renal fibrosis group (n = 5 per group). **(C)** RT-qPCR showed the different expression levels of the six key IRGs between the control samples and RIF samples with renal stone model (n = 5 per group). *, *P* < 0.05; **, *P* < 0.01; ***, *P* < 0.001; DEGs, differentially expressed genes; HE, hematoxylin and eosin; IRGs, immune-related DEGs.

Next, we analyzed the different expression levels of the six key IRGs between the RIF and control samples in the GSE12682 dataset and GSE76882 dataset (**Figure 2E**). The results from both indicated that the expression levels of APOH and EGF in the fibrosis group were significantly lower than those in the control group, while the expression levels of LTF, LYZ, PLTP, and SLPI were higher (all *P* < 0.05), which was consistent with the six sets of DEGs in CKD samples of six microarray datasets and the renal fibrosis samples in the GSE38117 dataset of UUO mice model (**Supplementary Figure 2**). Importantly, we collected the renal samples from the stones model group *in vivo*, which verified kidney injury and RIF in the stones model group by HE staining and Sirius Red staining (**Figures 3A, B**), and found same pattern of six gene expression changes using RT-qPCR (**Figure 3C**). In addition, the correlation analysis suggested a significantly strong or moderate correlation among the six key markers (all *P* < 0.05) (**Figure 2F**, **Supplementary Table 4**).

### Validation of immune-correlated key biomarkers

**Figure 4:**
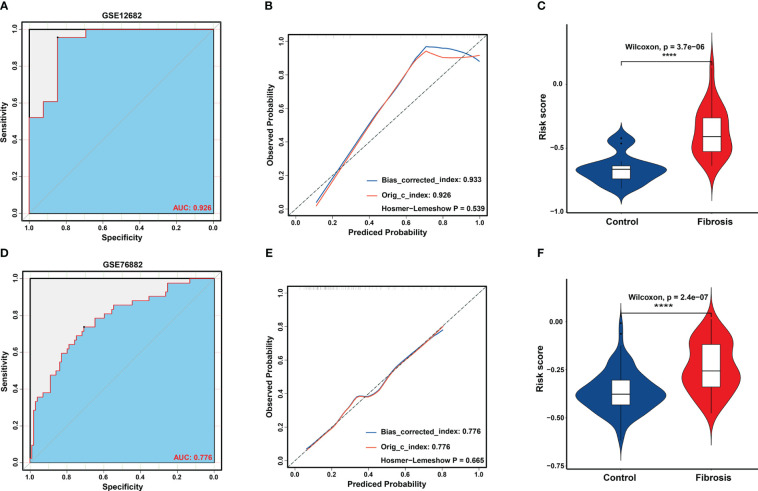
Validation of key biomarkers for RIF. **(A, D)** ROC analysis revealed good diagnostic performance of the risk score for RIF among the GSE12682 datasets and GSE76882 dataset. **(B, E)** Calibration plot of the risk score for predicting the probability of RIF among the GSE12682 datasets and GSE76882 dataset. **(C, F)** Violin plots of risk scores between the RIF and control groups among the GSE12682 datasets and GSE76882 dataset. ****, *P* < 0.0001.

Based on the LASSO regression model, the risk score was calculated for each sample with the expression value of genes and corresponding coefficients: Risk score = [(-0.05627) × Expression value of APOH] + [(-0.00264× Expression value of EGF] + [(0.03986) × Expression value of LTF] + [(0.02047) × Expression value of LYZ] + [(0.02807) × Expression value of PLTP] + [(-0.06040) × Expression value of SLPI]. Subsequently, ROC analysis and C-index analysis were applied to evaluate the diagnostic value of the six-gene risk scores. A favorable diagnostic efficacy of the six-gene risk scores in discriminating RIF from control samples, with an AUC of 0.926, was found in the GSE12682 dataset, and the C-index of the risk score was 0.933 (**Figures 4A, B**). Moreover, the risk score showed a powerful diagnostic ability in the GSE76882 dataset, with an AUC of 0.776 and C-index of 0.776 (**Figures 4D, E**). The fibrosis samples obtained significantly higher risk scores than the control samples in both the GSE12682 and GSE76882 datasets (all *P* < 0.0001) (**Figures 4C, F**).

**Figure 5:**
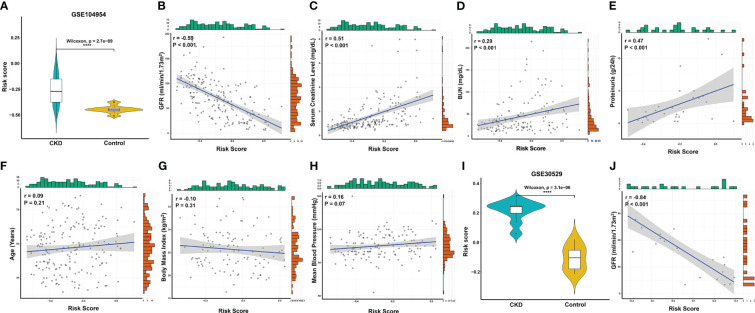
Correlation analysis between the risk score and clinical features in CKD patients. **(A)** Violin plots of risk scores between the CKD and control groups from the GSE104954 dataset. **(B–E)** Significantly correlation between the risk score and GFR **(B)**, serum creatinine level **(C)**, BUN **(D)**, and proteinuria **(E)** in CKD patients from the GSE104954 dataset. **(F–H)** The correlation between risk score and age **(F)**, body mass index **(G)**, and mean blood pressure **(H)** in CKD patients from the GSE104954 dataset. **(I)** Violin plots of the risk score between the CKD and control groups from the GSE30529 dataset. **(J)** Significantly negative correlation between the risk score and GFR in CKD patients from the GSE30529 dataset. ****, *P* < 0.0001. GFR, glomerular filtration rate; BUN, blood urea nitrogen; CKD, chronic kidney disease.

Given that RIF is often accompanied by renal dysfunction, we explored the correlation between the risk score and clinical characteristics in the GSE104954 dataset and GSE30529 dataset, which included CKD samples with different etiologies that caused RIF, such as DN, MCD, HT, IgA nephropathy, TMD, FSGS, MGN, RPGN, and SLE (**Supplementary Table 5**) (6, 12). Similar to the results in the GSE12682 dataset, the risk scores in the CKD samples were significantly higher than those in the control samples (all *P* < 0.0001) (**Figures 5A, I**). Moreover, the risk score was strongly negatively correlated with GFR (GSE104954: r = -0.59, *P* < 0.0001; GSE30529: r = -0.84, *P* < 0.0001) (**Figures 5B, J**) and positively correlated with serum creatine level (r = 0.51, *P* < 0.0001) (**Figure 5C**), BUN level (r = 0.29, *P* < 0.0001) (**Figure 5D**), and proteinuria (r = 0.47, *P* < 0.001) (**Figure 5E**). No significant correlation was found with age (**Figure 5F**), body mass index (**Figure 5G**), or mean blood pressure (**Figure 5H**) (all *P* > 0.05).

### Functional enrichment analysis of the fibrosis samples

**Figure 6:**
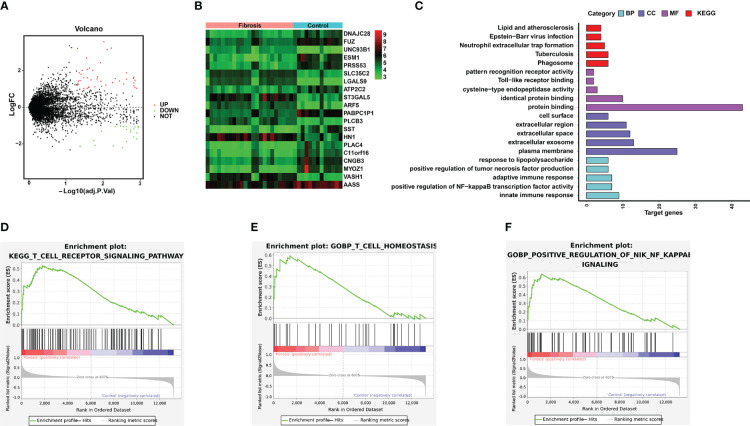
Significant pathways associated with the DEGs between the RIF and control samples in the GSE12682 dataset. **(A)** Volcano plot of the GSE12682 dataset. A total of 43 upregulated DEGs and 21 downregulated DEGs were identified between the RIF and control samples. **(B)** Heatmap of DEGs between the RIF and control samples in the GSE12682 dataset. **(C)** DEGs with their top 5 enriched GO terms and KEGG terms. **(D–F)** GSEA for the GSE12682 dataset.

To better understand the biological information associated with RIF, we obtained the DEGs between the RIF and control samples in the GSE12682 dataset. A total of 43 upregulated DEGs and 21 downregulated DEGs were identified, and their different expression patterns were visualized using volcano plots and heatmaps (**Figures 6A, B**). Then, GO enrichment analysis and KEGG analysis were performed using the online DAVID tool. The results indicated a strong association with the adaptive innate immune response, innate immune response, and positive regulation of NF-κB transcription factor activity (**Figure 6C**, **Supplementary Table 6**). Moreover, GSEA was performed between the fibrosis and control samples in the GSE12682 dataset. Several immune pathways involved in fibrosis, such as the “T cell receptor signaling pathway” (NES, 1.688, NOM *p*-value < 0.001), “T cell homeostasis” (NES, 1.624, NOM *p*-value = 0.008), and “positive regulation of NF-κB signaling pathway” (NES, 1.756, NOM *p*-value < 0.001) were identified (**Figures 6D–F**, **Supplementary Table 7**).

### Enrichment of immune cells in the fibrosis and control samples

**Figure 7:**
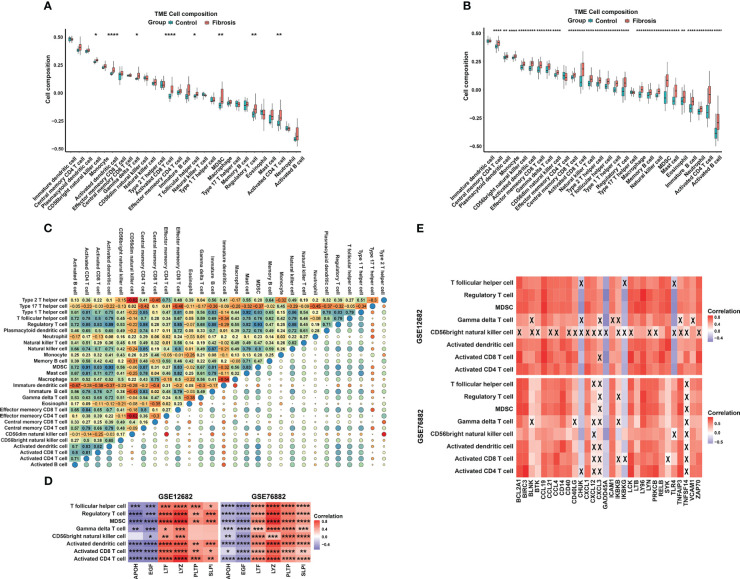
The distribution of 28 types of immune cells between the RIF and control samples. **(A, B)** Violin plots of 28 types of immune cells that were differentially enriched in the **(A)** GSE12682 dataset and **(B)** GSE76882 dataset. **(C)** Heatmap of the correlation of 28 types of immune cells in the GSE12682 dataset. The size of the colored squares represents the strength of the correlation. Darker color implies a stronger association. **(D)** Heatmap of correlations among 8 types of immune cells and the six key IRGs in the GSE12682 dataset and GSE76882 dataset. **(E)** Heatmap of correlations among the genes from the NF-κB signaling pathway and 8 types of immune cells in the GSE12682 dataset and GSE76882 dataset. *, *P* < 0.05; **, *P* < 0.01; ***, *P* < 0.001; ****, *P* < 0.0001; X, *P* > 0.05.

To explore the difference in the abundances of immune cell subtypes between the fibrosis and control groups, 28 available immune cell subtypes were assessed in the GSE12682 dataset using the ssGSVA method. The results indicated that 8 types of immune cells (including T follicular helper cells, Treg cells, MDSCs, gamma delta T cells, CD56bright natural killer cells, activated CD4 T cells, activated CD8 T cells, and activated dendritic cells) were significantly enriched in a higher proportion as in the control group (all *P* < 0.05) (**Figure 7A**). Moreover, the GSE76882 dataset showed similar phenotypes of change (all *P* < 0.05) (**Figure 7B**). The interrelation among the various immune cell subtypes in the GSE12682 dataset varied from weak to moderate (**Figure 7C**). Next, we conducted correlation analyses to explore the relationship between the six key IRGs and different immune cell types (**Supplementary Figure 3**). As shown in **Figure 7D**, all six key markers showed a significantly strong correlation with six types of immune cells (all *P* < 0.05), except for gamma delta T cells and CD56bright natural killer cells. Because the NF-κB signaling pathway was associated with the progression of fibrosis, we also performed correlation analyses between eight immune cells and the genes in the NF-κB signaling pathway from the KEGG database. As shown in **Figure 7E** and **Supplementary Figure 4**, most immune cells were significantly associated with genes in the NF-κB signaling pathway, especially genes that activate the T-cell signaling pathway and noncanonical pathway (**Supplementary Table 8**).

### Involvement of key markers in DC cells and T cells

**Figure 8:**
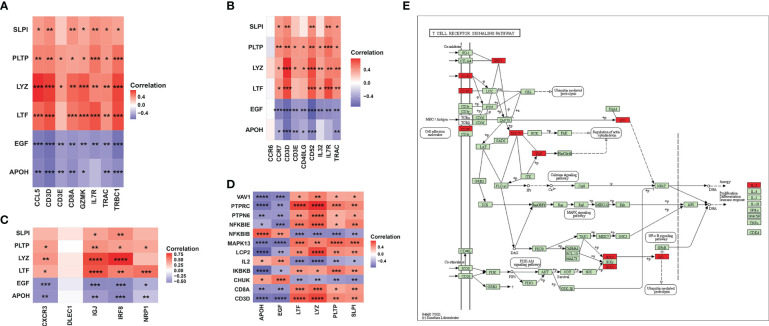
Association between six key IRGs and immune cell markers. **(A)** Correlation between the expression levels of the six IRGs and CD8+ T-cell markers. **(B)** Correlation between the expression levels of the six IRGs and T helper cell markers. **(C)** Correlation between the expression levels of the six IRGs and DC markers. **(D)** Correlation between the expression levels of the six IRGs and the genes in the T-cell receptor signaling pathway. **(E)** Regulatory network of the T-cell receptor signaling pathway as obtained from the KEGG database. The red square indicates that the gene was positively correlated with the IRGs, while the purple square indicates that the gene was negatively correlated with the IRGs. *, *P* < 0.05; **, *P* < 0.01; ***, *P* < 0.001; ****, *P* < 0.0001.

To further analyze the correlation between key markers and DC cells or T cells, we obtained gene sets related to CD8+ T-cell markers, T helper cell, and DC cell markers in human kidney from CellMarker and applied Pearson correlation analysis among the six IRGs and three gene sets in the GSE12682 dataset (**Figures 8A–C**, **Supplementary Table 9**). The results suggested that six IRGs were strongly correlated with most genes related to three immune cell markers (all *P* < 0.05), except for DLEC1 as a DC cell marker and CCR6 as a T helper cell marker (all *P* > 0.05). We also explored the correlation of six IRGs with the genes in the T-cell receptor signaling pathway (**Supplementary Table 9**). As shown in **Figures 8D, E**, all key markers were highly associated with the genes in the cell adhesion molecules (PTPRC, CD8A, CD3D, all *P* < 0.05), PI3K-Akt signaling pathway (IKBKB and CHUK, all *P* < 0.05), and NF-κB signaling pathway (NFKBIE and NFKBIB, all *P* < 0.05). These results indicate that T cells and DCs play an important role in regulating the process of RIF.

## Discussion

As the third-leading cause of premature mortality, CKD has been a major worldwide public disease burden (18). At the histological level, renal fibrosis, particularly renal interstitial fibrosis (RIF), is the ultimate common pathway of progressive kidney disease, no matter what the initial injury is (12). Importantly, immune-related genes play critical roles in the initiation and progression of renal fibrosis (19). Therefore, it is of great clinical significance to identify immune-related biomarkers and potential immune cell infiltration changes for the intervention against RIF to ultimately improve the prognosis of CKD.

In the present study, we screened a total of 928 DEGs between CKD and control renal interstitial samples from the six microarray datasets and identified 17 overlapping immune-correlated DEGs after integration with the ImmPort database. Then, 6 IRGs, including 2 downregulated genes (APOH and EGF) and 4 upregulated genes (LTF, LYZ, PLTP, and SLPI) in CKD, were selected by LASSO regression analysis, validated as key biomarkers of RIF through ROC analysis in the GSE12682 dataset and GSE76882 dataset, and found to be significantly associated with renal function damage. GO enrichment analysis and GSEA indicated a strong association with the inflammatory response and T-cell receptor signaling pathway, as well as positive regulation of the NF-κB signaling pathway, for the DEGs between RIF and control samples in the GSE12682 dataset. Next, we found that various immune cells, especially T cells (including activated CD4 T cells, activated CD8 T cells, Treg cells, and T follicular helper cells), were significantly enriched in the RIF samples, which is similar to the change noted for the four upregulated IRGs and contrary to that noted for the two downregulated IRGs. Importantly, the 6 IRGs were not only strongly correlated with those immune cell markers but also might interact with genes in the T-cell signaling pathway, including the NF-κB signaling pathway and PI3K-Akt signaling pathway. Furthermore, the enriched immune cells were correlated with genes in the NF-κB signaling pathway. All of the above evidence indicated that the biological function of the 6 IRGs might involve the immune response mediated by the NF-κB signaling pathway and promote the progression of RIF.

RIF is considered to be a failed wound healing process that develops after chronic kidney damage from various insults (20). Initially, peritubular infiltration of numerous types of immune cells, particularly macrophages and T cells, is an early microenvironmental change that induces an inflammatory response and establishes a fibrogenic stage (21). Subsequently, myofibroblast activation and expansion, followed by EMT and cell apoptosis, result in tubular atrophy, impairment of renal function, and finally end-stage renal disease. Therefore, inflammation has an important role in the initiation and progression of renal fibrogenesis after injury (18, 22). Considerable evidence has shown that CD4+ T cells, Th17 cells and gamma delta T cells exert a profibrotic effect on damaged kidneys, whereas Tregs prevent kidney injury and fibrosis (23–25). Our results indicated that the infiltration of many immune cells, especially T cells with various functions (including activated CD4 T cells, activated CD8 T cells, T helper cells, and regulatory T cells) and activated DCs, was significantly increased in the RIF samples, probably contributing to RIF occurrence and progression. Although Treg cells function as anti-fibrotic immune cells, a previous study indicated that Treg cells are an important population that preferentially accumulates in fibrotic mouse kidneys (24), which is consistent with our results. Moreover, prophylactic Treg expansion confers protection against kidney injury and fibrosis development (24). Notably, CD8+ T cells have opposite roles in renal inflammation and fibrosis. A previous study found that CD8+ T cells accumulated early in the renal interstitium, reaching a peak at Day 5 in a unilateral ureter obstruction (UUO) model of renal fibrosis (26). CD8+ T cells induce M1/M2 macrophage polarization to promote a stronger inflammatory response and facilitate the proliferation and activation of resident myofibroblasts (27, 28). However, recent studies indicated that increased infiltration of CD8+ T cells restrains renal fibrosis, and CD8+ T-cell deficiency aggravates renal fibrosis in UUO-treated mice (26, 29). Therefore, immune cells play an important role in the process of renal fibrosis, but how the various immune cells induce inflammatory responses and the specific mechanisms involved in the progression of fibrosis remain to be verified.

As the initial stage of renal fibrosis, uncontrolled or excessive inflammation is bound to cause progressive renal injury in the context of CKD. We screened 6 sets of DEGs from multiple CDK renal interstitial tissues, selected immune-related DEGs, and identified 6 IRGs related to RIF. Our results demonstrated that several signaling pathways, such as the “T cell receptor signaling pathway” and “positive regulation of NF-κB signaling pathway”, were involved in the process of RIF, and the 6 IRG expression levels were significantly correlated with the genes in these signaling pathways. Activation of the NF‐κB pathway stimulates a proinflammatory response and promotes renal fibrosis, and treatment with an NF-κB inhibitor attenuates renal injury and inflammation in CKD tissues (30–32). In a UUO model of progressive kidney disease for mice that express the human CRP gene (CRPtg), severe renal inflammation and fibrosis with a significant increase in tubulointerstitial T cells and macrophages have been found, accompanied by increased activation of both the NF-κB/p65 and TGF-β/Smad2/3 signaling pathways (33). In addition, a previous study found that CXCL16 plays a role in angiotensin II-induced renal inflammation and RIF in tubular epithelial cells in an NF-κB-dependent manner, while CXCL16 deficiency inhibited the infiltration of F4/80+ macrophages and CD3+ T cells in the kidney (34). These data indicate that activation of NF-κB signaling and infiltration of T cells might co-occur during fibrosis. However, it has not been determined how this coexpression phenotype is induced. We provide correlation analysis evidence that the 6 IRGs were strongly correlated with the genes in the NK-κB signaling pathway, which positively regulates the T-cell signaling pathway. Moreover, the four upregulated IRGs (LTF, LYZ, PLTP, SLPI) were positively associated with various T cell populations, which were enriched in RIF tissues, whereas the two downregulated IRGs (APOH and EGF) had opposite results. Therefore, one reasonable hypothesis is that these IRGs induce inflammatory responses and promote RIF by mediating the NF-κB signaling pathway in T cells, ultimately impairing renal function.

Six key IRGs, including APOH, EGF, LTF, LYZ, PLTP, and SLPI, were identified to be associated with RIF. APOH, also known as β_2_-glycoprotein 1 (β2GPI), is the most common protein for antiphospholipid antibodies in chronic disorders related to endothelial cell dysfunction (35). APOH was identified as a complement regulator and mediates innate immune regulation (36). Oxidized APOH causes DCs to mature and primes naive T lymphocytes, thus inducing T helper 1 (Th1) polarization, which involves NF-κB activation and interleukin-1 receptor associated kinase (IRAK) phosphorylation (35, 37). In a model of STZ-DN mice, exogenously administered purified β2GPI decreased the expression levels of TGF-β1 and collagen IV, with concomitant inhibition of p38 MAPK, and thus exerted renoprotective and antifibrotic effects (38). EGF, as a protein that stimulates cell growth and differentiation, was found to be expressed at low levels in the urine of end-stage kidney disease cases and strongly correlated with eGFR and urine albumin-to-creatinine ratio (39). A previous study indicated that diminished EGF levels lead to the development of kidney fibrosis associated with renal β-catenin/mTOR hyperactivation and predispose kidneys to progressive renal disease (40). Considerable evidence has shown that LTF and LYZ have important antioxidant, anti-inflammatory and nephroprotective activities (41–43). LTF inhibits TGF-β1-induced renal fibrosis by restraining the expression of the profibrogenic genes CTGF, PAI-1 and collagen I (44). PLTP not only influences lipid transfer and lipoprotein metabolism, which is associated with cardio-metabolic diseases (45), but also plays a key role in the modulation of adaptive immune functions through alternation of T cell helper polarization (46). Similarly, the urinary levels of PLTP and SLPI are both increased in CKD patients with renal fibrosis (47, 48). SLPI is a critical mediator that controls anabolic parathyroid hormone-induced bone formation (49). As an inhibiting proteolytic enzyme, SLPI involves in immune functions of MSC to control T-cell proliferation and the regulation of damaged tissue healing, and was also identified as an ideal biomarker for kidney injury (50, 51).

Unfortunately, several limitations in the present study cannot be ignored. First, our results indicated that 6 IRGs were significantly correlated with renal function damage in CKD populations, but clinical information was not available for the RIF samples in the GSE12682 and GSE76882 datasets. Importantly, the multiple datasets included in the analysis did not provide detailed data on the extent of fibrosis in the RIF samples. Therefore, whether the 6 IRGs play an initiating or continuing role in the fibrosis process remains unclear. Second, due to the limited research conditions, this retrospective study was performed based on microarray datasets and *in vivo* experiments. Therefore, direct evidence, such as clinical information and human RIF patient samples, is needed to uncover the potential pathophysiological mechanisms of these IRGs in the progression of RIF. Third, the immune cell infiltration in RIF samples was inferred by the ssGSEA method. These findings may deviate from the heterotypic interactions of cells, disease-induced disorders, or phenotypic plasticity, a shortcoming that needs to be addressed by further studies. A recent study demonstrated an increase in the number of infiltrating CD45^+^ cells and CD45^+^/CD3^+^ T cells in renal specimens from UUO mice with RIF. These research findings support our bioinformatics analyses regarding changes in immune cell infiltration (52). Undoubtedly, we will spare no effort to further explore the mechanisms of RIF in CKD populations.

## Conclusion

In summary, six IRGs (APOH, EGF, LTF, LYZ, PLTP, and SLPI) were identified as key biomarkers for RIF. These IRGs exhibited a strong correlation with various T cells, activated DCs, and with the NF-κB signaling pathway. All these IRGs and their signaling pathways may evolve as valuable therapeutic targets for RIF in CKD.

## Data availability statement

The original contributions presented in the study are included in the article/**Supplementary Materials**, further inquiries can be directed to the corresponding author/s.

## Ethics statement

The animal study was approved by the Ethics Committee for Animal Research of the Xiangya hospital of Central South University (202301003). The study was conducted in accordance with the local legislation and institutional requirements.

## Author contributions

ZTD and XC supervised the project, designed the study, and interpreted the data. FZC and XC performed data management and analyzed the data. SP, XFL, XYL, and LZG took part in analyzing the data. XC wrote the first draft of the manuscript. ZTD and EW wrote and reviewed the manuscript. All of the authors approved the final version of the manuscript.

## Supplementary material

Supplementary Figure 1 Identification of DEGs associated with CKD. (A) Volcano plot (left) and heatmap (right) of the GSE30529 dataset. In total, 416 DEGs were identified from the GSE30529 dataset in the CKD samples vs. the control, including 298 upregulated and 118 downregulated genes. (B) Volcano plot (left) and heatmap (right) of the GSE32591 dataset. In total, 125 DEGs were identified from the GSE32591 dataset, including 97 upregulated and 28 downregulated genes. (C) Volcano plot (left) and heatmap (right) of the GSE35487 dataset. In total, 36 DEGs were identified from the GSE35487 dataset, including 36 downregulated and 0 upregulated genes. (D) Volcano plot (left) and heatmap (right) of the GSE37455 dataset. In total, 27 DEGs were identified from the GSE37455 dataset, including 10 upregulated and 17 downregulated genes. (E) Volcano plot (left) and heatmap (right) of the GSE121211 dataset. In total, 78 DEGs were identified from the GSE121211 dataset, including 49 upregulated and 29 downregulated genes. (F) Volcano plot (left) and heatmap (right) of the GSE133288 dataset. In total, 246 DEGs were identified from the GSE133288 dataset, including 198 downregulated and 48 upregulated genes. DEGs, differentially expressed genes; CKD, chronic kidney disease.

Supplementary Figure 2 The expression levels of the six key IRGs in the GSE38117 dataset. Violin plot illustrating the gene expression profile of three fibrotic kidneys with three undamaged contralateral kidneys in mice using the UUO model. *, *P* < 0.05, the paired Student’s t-test.

Supplementary Figure 3 Correlation between the expression levels of six key IRGs and immune cell enrichment. (A-F) Correlation between the expression levels of APOH (A), EGF (B), LTF (C), LYZ (D), PLTP (E), and SLPI (F) and 28 enriched types of immune cells in the GSE12682 dataset. The size of each dot represents the strength of the correlation between the IRG and the immune cell. The color of the dot represents the *P* value; the greener the color, the higher the *P* value, and the redder the color, the lower the *P* value. *P* < 0.05 was considered statistically significant.

Supplementary Figure 4 Correlation between the infiltration of immune cells and genes in the NK-κB signaling pathway.
