# Supplementary figures and images for: Identification of the key immune-related genes and immune cell infiltration changes in renal interstitial fibrosis
Source: Front Endocrinol (Lausanne). 2023 Nov 8;14:1207444. doi: 10.3389/fendo.2023.1207444 (PMC10663291; doi:10.3389/fendo.2023.1207444)

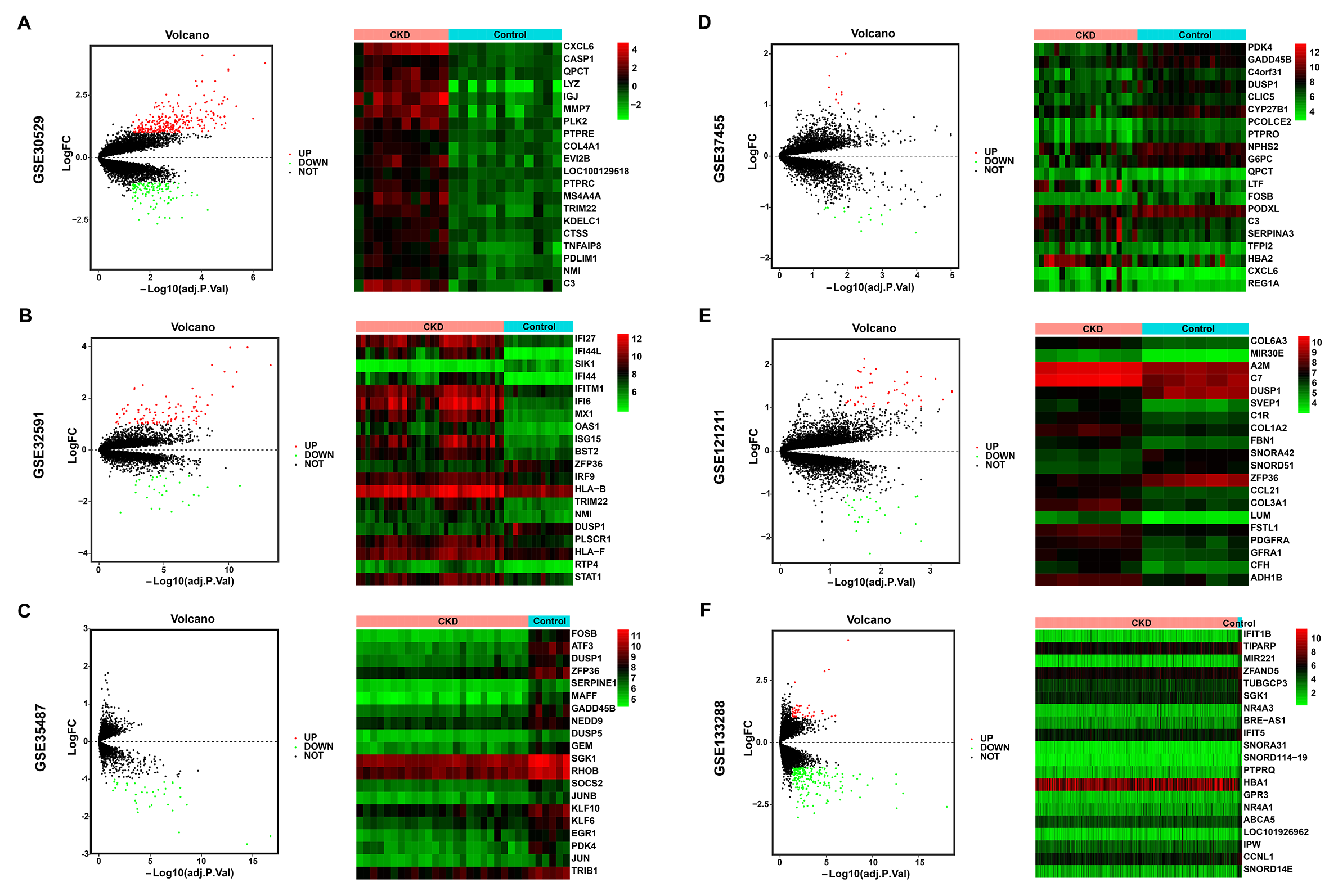

Supplement: Supplementary Figure 1 — Identification of DEGs associated with CKD. (A) Volcano plot (left) and heatmap (right) of the GSE30529 dataset. In total, 416 DEGs were identified from the GSE30529 dataset in the CKD samples vs. the control, including 298 upregulated and 118 downregulated genes. (B) Volcano plot (left) and heatmap (right) of the GSE32591 dataset. In total, 125 DEGs were identified from the GSE32591 dataset, including 97 upregulated and 28 downregulated genes. (C) Volcano plot (left) and heatmap (right) of the GSE35487 dataset. In total, 36 DEGs were identified from the GSE35487 dataset, including 36 downregulated and 0 upregulated genes. (D) Volcano plot (left) and heatmap (right) of the GSE37455 dataset. In total, 27 DEGs were identified from the GSE37455 dataset, including 10 upregulated and 17 downregulated genes. (E) Volcano plot (left) and heatmap (right) of the GSE121211 dataset. In total, 78 DEGs were identified from the GSE121211 dataset, including 49 upregulated and 29 downregulated genes. (F) Volcano plot (left) and heatmap (right) of the GSE133288 dataset. In total, 246 DEGs were identified from the GSE133288 dataset, including 198 downregulated and 48 upregulated genes. DEGs, differentially expressed genes; CKD, chronic kidney disease. [file Image_1.tif]

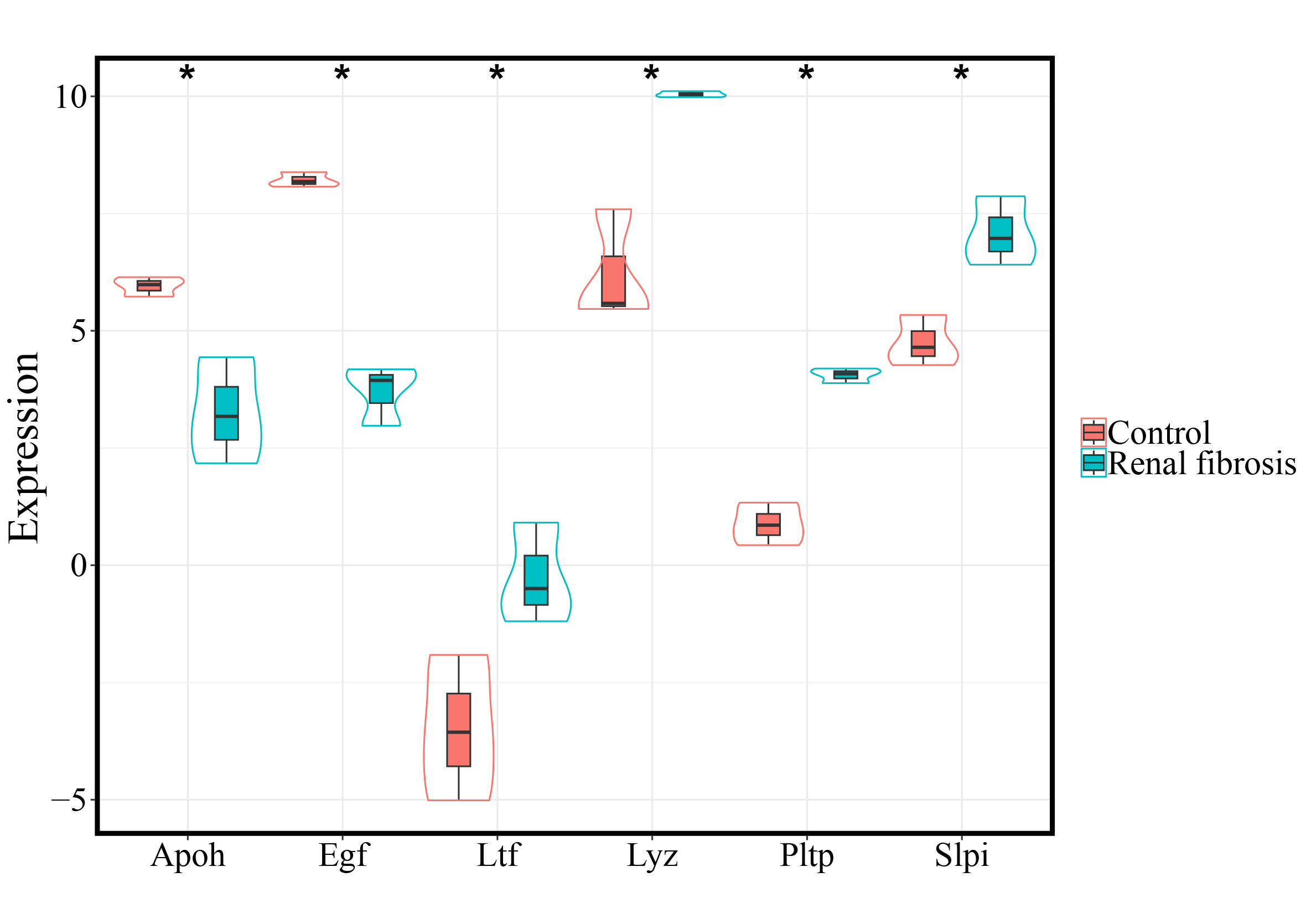

Supplement: Supplementary Figure 2 — The expression levels of the six key IRGs in the GSE38117 dataset. Violin plot illustrating the gene expression profile of three fibrotic kidneys with three undamaged contralateral kidneys in mice using the UUO model. *, P < 0.05, the paired Student’s t-test. [file Image_2.tif]

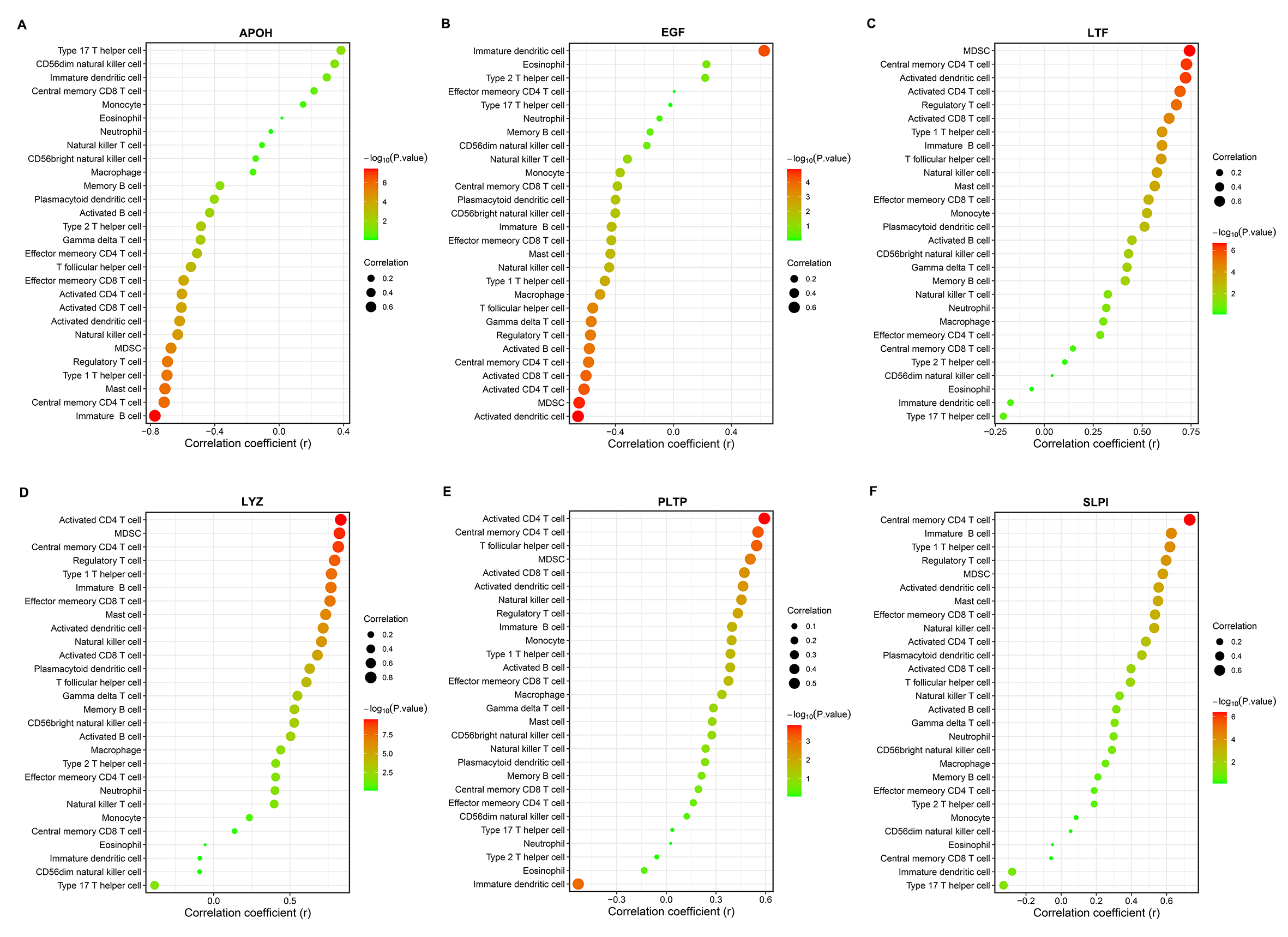

Supplement: Supplementary Figure 3 — Correlation between the expression levels of six key IRGs and immune cell enrichment. (A-F) Correlation between the expression levels of APOH (A), EGF (B), LTF (C), LYZ (D), PLTP (E), and SLPI (F) and 28 enriched types of immune cells in the GSE12682 dataset. The size of each dot represents the strength of the correlation between the IRG and the immune cell. The color of the dot represents the P value; the greener the color, the higher the P value, and the redder the color, the lower the P value. P < 0.05 was considered statistically significant. [file Image_3.tif]

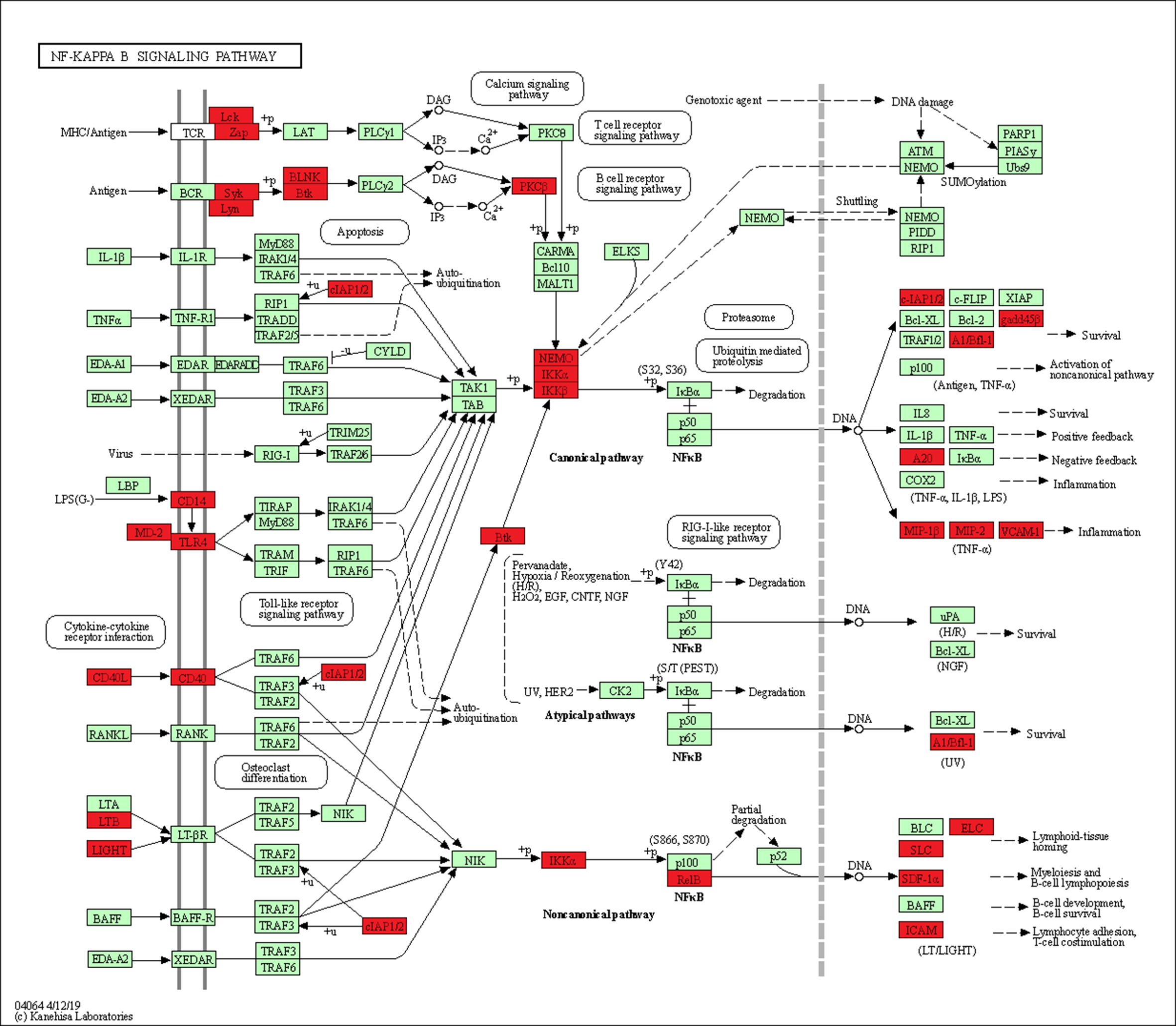

Supplement: Supplementary Figure 4 — Correlation between the infiltration of immune cells and genes in the NK-κB signaling pathway. [file Image_4.tif]
