# Supplementary material for: Identification of the key immune-related genes and immune cell infiltration changes in renal interstitial fibrosis
Source: Front Endocrinol (Lausanne). 2023 Nov 8;14:1207444. doi: 10.3389/fendo.2023.1207444 (PMC10663291; doi:10.3389/fendo.2023.1207444)
Supplement: Supplementary file 5 [file Table_1.docx]

Supplementary Table 1. Characteristics of the individual studies.

| **Datasets** | **Characteristics** | **No.** | **Organism** | **Series type** | **Series platform ID** |
| --- | --- | --- | --- | --- | --- |
| GSE30529 | 10 DN and 12 healthy controls | 22 | Homo sapiens | Expression profiling by array | GPL571 |
| GSE35487 | 25 IgAN and 6 healthy controls | 31 | Homo sapiens | Expression profiling by array | GPL96 |
| GSE37455 | 20 HTN and 21 healthy controls | 41 | Homo sapiens | Expression profiling by array | GPL14663 |
| GSE133288 | 69 FSGS, 54 MCD, 48 MN, 68 IgAN, and 5 healthy controls | 244 | Homo sapiens | Expression profiling by array | GPL19983 |
| GSE121211 | 5 FSGS and 5 healthy controls | 10 | Homo sapiens | Expression profiling by array | GPL17586 |
| GSE32591 | 32 SLE and 15 healthy controls | 47 | Homo sapiens | Expression profiling by array | GPL14663 |
| GSE12682 | 23 CKD and 13 healthy controls | 36 | Homo sapiens | Expression profiling by array | GPL571 |
| GSE76882 | 42 IFTA and 99 healthy controls | 141 | Homo sapiens | Expression profiling by array | GPL13158 |
| GSE38117 | 3 left UUO and 3 right healthy kidney controls | 3 | Mus musculus | Expression profiling by array | GPL4134 |

DN, diabetic nephropathy; IgAN, IgA nephropathy; HTN, hypertensive nephropathy; FSGS, focal and segmental glomerulosclerosis; MCD, minimal change disease; MN, membranous nephropathy; SLE, systemic lupus erythematosus; CKD, chronic kidney disease; IFTA, interstitial fibrosis and tubular atrophy; UUO, ureteral unilateral obstruction.
